# A Novel Method for Constructing a WIFI Positioning System with Efficient Manpower

**DOI:** 10.3390/s150408358

**Published:** 2015-04-10

**Authors:** Yuanfeng Du, Dongkai Yang, Chundi Xiu

**Affiliations:** School of Electronic and Information Engineering, Beihang University, Beijing 100191, China; E-Mails: edkyang@buaa.edu.cn (D.Y.); xcd@buaa.edu.cn (C.X.)

**Keywords:** location-based service, Radio Fingerprinting Database, RWGR, WIFI positioning

## Abstract

With the rapid development of WIFI technology, WIFI-based indoor positioning technology has been widely studied for location-based services. To solve the problems related to the signal strength database adopted in the widely used fingerprint positioning technology, we first introduce a new system framework in this paper, which includes a modified AP firmware and some cheap self-made WIFI sensor anchors. The periodically scanned reports regarding the neighboring APs and sensor anchors are sent to the positioning server and serve as the calibration points. Besides the calculation of correlations between the target points and the neighboring calibration points, we take full advantage of the important but easily overlooked feature that the signal attenuation model varies in different regions in the regression algorithm to get more accurate results. Thus, a novel method called RSSI Geography Weighted Regression (RGWR) is proposed to solve the fingerprint database construction problem. The average error of all the calibration points’ self-localization results will help to make the final decision of whether the database is the latest or has to be updated automatically. The effects of anchors on system performance are further researched to conclude that the anchors should be deployed at the locations that stand for the features of RSSI distributions. The proposed system is convenient for the establishment of practical positioning system and extensive experiments have been performed to validate that the proposed method is robust and manpower efficient.

## 1. Introduction

Nowadays people are more and more concerned about their precise location information for the wide use of location-based services in daily life. Global Navigation Satellite System (GNSS), as an effective way of outdoor positioning, has become a requisite tool for traveling. Unfortunately, for indoor scenarios, the satellite signal is disrupted or blocked and a person would be located with poor accuracy, or even unable to be located. According to social scientists, indoor positioning is highly desirable since 70% of daily life is indoors. Consequently, several indoor positioning technologies have emerged, such as infrared-based positioning, ultra-wideband (UWB) positioning, radio frequency identification (RFID) positioning, and WIFI positioning, most of which are too costly to be used in large areas.

With the development of wireless networks, a large amount of WIFI APs have been installed, which represents a convenient foundation for WIFI positioning methods. In addition to the availability, a unique advantage of WIFI positioning technology is that most kinds of mobile devices are already equipped with WIFI modules. Among the various WIFI positioning systems, the fingerprint technology, in which the user’s location is estimated by matching online received signal strength index (RSSI) with values collected offline, is one of the most feasible approaches [1,2].

In recent years, the WIFI-based localization systems have shown great promise and researchers have focused on several key challenges in real-time accuracy fingerprint positioning for practical deployment. For example, solutions to deal with the heterogeneity of WIFI devices have been addressed in [3,4]. Several studies have investigated noise and multipath distortion to improve the accuracy and robustness [5,6]. The radio mismatch problems such as the different user orientations have been studied in [7]. Besides the static positioning approach, many researchers have proposed methods to combine WIFI positioning systems with sensors for tracking, including gyroscopes, accelerometers and magnetometers [8,9]. What’s more, the indoor map matching technology can be integrated in the WIFI positioning systems [10].

However, for practical applications, the biggest issue of WIFI indoor positioning is the construction and updating of the radio fingerprint database, which requires professional signal collection, and is both time- and labor-consuming, especially in large urban areas. Due to the fluctuating feature of the WIFI signals, the database should be updated periodically by workers, even for the same scenario. Features of the signal are obviously different between office hours and rush hours, and dramatic positioning errors would be generated if the same fingerprint database is adopted. More seriously, the signal environment may change, and APs may be displaced or upgraded, which could also greatly affect the positioning accuracy. Therefore, the radio map for positioning should be constructed and updated adaptively [11].

It has been stated in [12] that a reduction of the manual efforts required for this task can be achieved by minimizing the sampling time at each reference point (RP) and/or by limiting the number of locations to sample from. Nevertheless, this simple idea produces inaccurate radio maps, which decrease the accuracy of the location estimation. Consequently, many other attempts have been proposed, including the free-calibration methods [13,14,15,16,17,18,19,20], user-aided methods [21,22] and the auto-update technologies [23].

In [13], a dynamic radio map is constructed to reduce the cost significantly and the real-time RSSI values at reference points are predicted based on the RSSI values at calibration points. Another system, QRLoc [14], automatically collects fingerprints when smartphone users scan Quick Response (QR) codes attached at known and fixed locations such as signs and posts. This method requires placing the QR codes in advance.

In [15], a novel approach, where the training data are obtained by means of finite-difference time-domain (FDTD) simulations of the electromagnetic propagation in the considered scenario, is presented. The performance of the method is assessed by means of experimental results in a real scenario. However, the great number of parameters of the environment as described in [15] should be provided for the signal simulations.

Besides the training signal path loss models [16], the interpolation and regression technologies [17,18,19] have also been widely used. A micro-cell-based map construction method is proposed in [20] to deal with the unstable RSSI and build a metropolitan-scale radio map efficiently. However, the proposed interpolation algorithm is not accurate since it is only based on the relative distance.

An autonomous and collaborative RSSI fingerprint collection and localization system based on mobile users is proposed in [21], who track their positions with inertial sensors and measure RSSI from the surrounding access points. As the intended offline training phase is cut off, the result from the inertial sensors may be inaccurate. A motion detector is also used in [22] to determine whether the device is being moved or stationary, and the asynchronous interval labeling method is introduced, with great scope limits.

Whereas the paper [23] proposed a WIFI radio map generation and update solution using manifold alignment methods based on the available information, including a propagation modeling simulator, a limited number of labeled calibration fingerprints, and many crowd-sourced unlabeled measurements. Though the manifold alignment has been widely used to transfer the RSSI fingerprints information of different devices or different time to improve the system performances, it requires an initial integrated and accurate radio map and the environmental change is not taken into account.

The traditional WIFI positioning system is based on the client-server model, in which the client periodically collects the RSSI information of surrounding APs. However, the latest research in [24] introduces a novel client/server-based system that modifies the AP firmware to scan the surrounding APs and to broadcasts the power pattern recording results over the free information elements of the beacon frame defined by the WLAN standards. The centralized computer server will periodically receive the information report, including AP’s own Media Access Control (MAC) and AP’s own location, neighboring APs’ MACs and RSSIs. The Gauss process regression (GPR) algorithm adopted in the paper requires surveying Log-distance model in advance, which is hard to be accurately obtained in the whole environment. Another big weakness is that the performance of that positioning system may seriously depend on the locations and density of the existing APs.

Although research involving the fingerprint radio map concept have achieved some progress, an effective method for constructing and updating the fingerprint database to improve the applicability of WIFI positioning technology is still desirable. Compared with the former papers or systems, our main contributions of this paper lie in the following aspects. Firstly, instead of taking the full function devices as the anchors in former papers, our proposed sensor anchors are designed with a single function to periodically broadcast the standard WIFI beacon frame through uplink to APs, which are very cheap and can work for more than one year with an ordinary battery supply. Secondly, this paper proposes novel regression metrics and algorithms for signal fingerprint database construction. We take full advantage of the important but easily overlooked feature that the signal attenuation model varies in different regions in the regression algorithm to get more accurate results. Thus, a novel method called RSSI Geography Weighted Regression (RGWR) is proposed to solve the fingerprint database construction problem. Furthermore, the average error of all the calibration points’ self-localization results will help to make the final decision of whether the database is latest or to be updated automatically. The proposed system is convenient for the establishment of practical positioning system and extensive experiments have been performed to validate and verify the robustness and effectiveness.

The remainder of this paper is organized as follows: the new constructing model together with the algorithms is introduced in Section 2. An improved WLAN positioning structure is introduced, including a self-made low-power WIFI anchor together with a renovated AP, with the function of scanning both of the neighboring APs and the proposed WIFI anchors in the coverage. Besides the calculation of correlations between the target points and the neighboring calibration points, we take full advantage of the often forgotten feature that the variation of the signal attenuation models in different regions in the regression algorithm. Thus, a novel RSSI Geography Weighted Regression (RGWR) algorithm for the radio map construction problem is presented in detail. After the discussion of the deployment of the WIFI anchors, a new approach to detect and solve the fingerprint database updating problems is introduced. Experimental results in actual environments are detailed in Section 3 and conclusions are given in Section 4.

## 2. Methodology

### 2.1. Proposed Model

**Figure 1:**
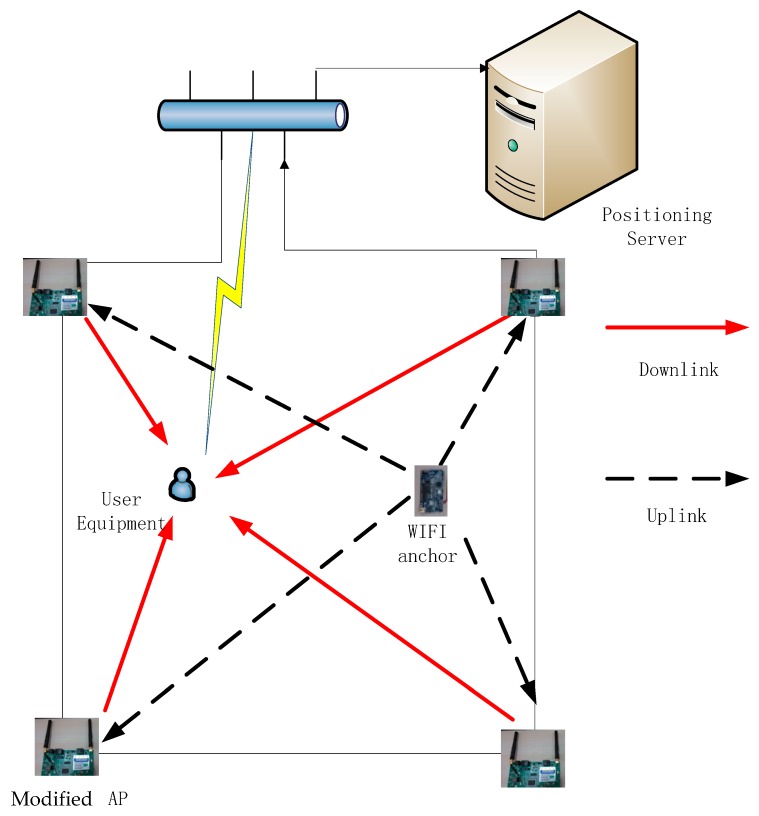
Improved WIFI positioning structure.

We first introduce an improved WLAN positioning structure in Figure 1, which includes a modified firmware and some cheap self-made WIFI anchors. Our AP is equipped with IEEE 802.11 WLAN transceiver hardware, so in addition to its default functionality as a wireless connectivity provider; it can also perform wireless scanning of both neighboring APs and our self-made WIFI anchors. Scanned online radio fingerprint recordings can be sent periodically to the centralized computer server together with their timestamps. WIFI anchors and APs are considered as calibration points with the real-time WIFI fingerprints and their locations available.

Note that the modified AP can be replaced by some wireless monitor equipment together with the traditional APs, and they are used here just for system prototyping and proof of concept. In the commercial implementation, the modified firmware is required to be adopted by an AP manufacturer and the power pattern recordings will be carried through the beacon frames to be received and decoded by any WIFI-enable device [24].

Generally, the user equipment (UE) receives the downlink signal from the APs. Inversely, the proposed WIFI anchor, called “uplink anchor”, can periodically broadcast the standard WIFI beacon frame through uplink to APs. The periodic dormancy mechanism with a designed duration of working and sleeping (e.g., 1 ms of signal transmission within every one second) is adopted, so that the anchor power consumption is little and can work for more than one year with the ordinary battery supply. By contrast, the anchor points proposed in [13,25] have to be equipped with a communication module to transfer the collecting data to the positioning server, with high cost and intensive system load. Although just using APs as calibration points like in [24] can construct and update a good fingerprint database, we still recommend using our proposed anchors in the system deployment to enhance the performance and robustness of database. APs are always deployed on the high walls with locations different from that of user mobile devices, and they are not very accurate to be used as calibration points. Our improvement by adding self-made anchors as calibration points is encouraging considering its low margin cost for practical deployment.

Based on the above-mentioned novel system framework, two fingerprint information tables can be obtained periodically. Table 1 is the online RSSI observation table between APs like the one in [24], and Table 2 is for each WIFI anchor.

**Table 1:** Online RSSI Observation between APs.

	$AP_{1}$	$AP_{2}$	$AP_{N}$
$Loc_{AP_{1}}$	—	$RSSI_{AP(1,2)}$	$RSSI_{AP(1,N)}$
$Loc_{AP_{2}}$	$RSSI_{AP(2,1)}$	—	$RSSI_{AP(2,N)}$
$Loc_{AP_{N}}$	$RSSI_{AP(N,1)}$	$RSSI_{AP(N,2)}$	—

**Table 2:** Online RSSI Observation for each anchor.

	$AP_{1}$	$AP_{2}$	$AP_{N}$
$Loc_{AC_{1}}$	$RSSI_{AC(1,1)}$	$RSSI_{AC(1,2)}$	$RSSI_{AC(1,N)}$
$Loc_{AC_{2}}$	$RSSI_{AC(2,1)}$	$RSSI_{AC(2,2)}$	$RSSI_{AC(2,N)}$
$Loc_{AC_{M}}$	$RSSI_{AC(M,1)}$	$RSSI_{AC(M,2)}$	$RSSI_{AC(M,M)}$

Here $RSSI_{AP}(i,j)$ is the RSSI fingerprint of $AP_{i}$ scanning by $AP_{j}$, and $RSSI_{AC}(i,j)$ ($RSSI_{A\text{nchor}}(i,j)$) is the RSSI fingerprint at the location of $Anchor_{i}$ scanning by $AP_{j}$. N is number of the APs（Access Points） and M is the number of the anchors. If one AP or WIFI anchor is not in the coverage of $AP_{j}$, the corresponding element in the table would be set to the smallest sensitivity (−92 dbm).

### 2.2. RGWR Algorithm

#### 2.2.1. Constructing Radio Map

From the proposed system, the WIFI radio map could be constructed based on $RSSI_{AP}$ and $RSSI_{AC}$. Since each AP can be considered independently, the construction process of the radio map for each AP is similar. Take $AP_{i}(x_{APi},y_{APi})$ for example. Given that the RSSI information of the APs $\left(RSSI_{AP}(1,i),RSSI_{AP}(2,i)\;...\;RSSI_{AP}(N,i)\right)$ and $\left(RSSI_{AC}(1,i),RSSI_{AC}(2,i)\;...\;RSSI_{AC}(M,i)\right)$ in the coverage of scanning, the question of how to construct the radio map can be transformed into how to calculate the signal strength in the whole area based on the calibration points. Many interpolation and regression algorithms have been proposed to solve this problem, such as the linear interpolation method [26], the Radial Basis Function (RBF) interpolation method [27], the GPR algorithm [24] and so on [26,27,28,29,30].

The traditional path loss models have proven that the attenuation is related to the distance and number of walls. Moreover, the research in [16] has demonstrated that the features of signal propagation in different regions can vary greatly. Motivated by the exiting research results and challenges, the principles we apply for the fingerprint database regression are as follows:

➢ The RSSIs among neighboring locations always exhibit some level of correlations.
➢ Signal attenuation models vary in different regions.

The first principal is adopted by most weighted regression algorithms. The second one, however, is always neglected.

In this paper, the RSSI Geography Weighed Regression (RGWR) method is proposed to construct the WIFI radio map for positioning, considering its characteristics fit our two principles well. The Geography Weighted Regression (GWR) is a special form of the vary-coefficient regression method, which considers that the regression coefficients are not only affected by the known anchors surrounding, but also vary as the distance from anchors and the located regions change [31]. GWR is used in the construction and updating of the fingerprint database for the first time as the authors’ knowledge.

To apply the RGWR method, available information includes the coordinate of each reference point, both of the AP and WIFI anchors, the distance between each anchor and the AP, and the signal strength received by each AP from Table 1 and Table 2.

The logarithmic-distance path loss model log(d) has been used for the received signal strength calculation. In addition, the transmission loss along a path accumulates as more walls are encountered, and the number of such walls tends to be proportional to the path length. Thus, the path loss model is improved in Equation (1), which adopts a third least-square fitting parameter [32]:

(1) $$PL=A+B\log(d/d_{0})+C\exp(\log(d/d_{0})/\log e)+S$$

where A, B, C are the parameters of the mean path loss PL of a certain point; and S is the variation of the mean and often referred to as shadow fading. d is the distance between the point and AP, $d_{0}=1\;m$ is the reference distance.

The following Equation (2) is the basic relationship, in which $Y_{i}$ stands for the RSSI value in the point *i*:

(2) $$RSSI_{i}=\beta_{0}(x_{i},y_{i})\text{+}\beta_{1}(x_{i},y_{i})X_{i1}+\beta_{2}(x_{i},y_{i})X_{i2}+\varepsilon_{i}$$

(3) $$\begin{array}{l} X_{i1}\text{=log}\left(d_{i}/d_{0}\right)\;\\ X_{i2}=\exp(\log(d_{i}/d_{0})/\log e)\; \end{array}$$

$\beta_{0}(x_{i},y_{i})$, $\beta_{1}(x_{i},y_{i})$ and $\beta_{2}(x_{i},y_{i})$ are the path loss coefficients. $d_{i}$ is the distance between the *i*th point and the AP. $\varepsilon_{i}$ is the measurement error of the *i*th point. e is the natural logarithm constant.

The estimator for this model is similar to the weighted least squares (WLS) model except that the weights are conditioned on the location $\left(x_{m},y_{m}\right)$ relative to the calibration anchors in the database and hence change for each location. The weights themselves are computed from a weighting scheme that is also known as a kernel. Many kinds of kernels are possible and a typical one has the Gaussian shape is adopted*,* which has been widely used to handle the WIFI RSSI [27]:

(4) wj(xm,ym)=e−0.5((xm-xj)2+(ym−yj)2)h)2 j=1,2,...,R

(5) $$W(x_{m},y_{m})=Diag(w_{1}(x_{m},y_{m}),w_{2}(x_{m},y_{m}),...,w_{R}(x_{m},y_{m}))$$

where $w_{j}(x_{m},y_{m})$ is the geographical weight of the jth calibration point in the dataset relative to the location $\left(x_{m},y_{m}\right)$. h is a parameter known as the width. $\left(x_{j},y_{j}\right)$ is the location of the jth calibration points. R is the total number of the calibration points, including the surrounding APs and WIFI anchors. Then, $W(x_{m},y_{m})$ is the weight matrix formed by the R points. Then, the regression coefficients $\beta (x_{m},y_{m})$ at sample location $\left(x_{m},y_{m}\right)$ can be obtained by the least mean-square error rule:

(6) $$\begin{array}{l} \beta (x_{m},y_{m})={\left(\beta_{0}(x_{m},y_{m}),\beta_{1}(x_{m},y_{m}),\beta_{2}(x_{m},y_{m})\right)}^{T}\\ \;={\left(X_{all}^{T}W(x_{m},y_{m})X_{all}\right)}^{-1}X_{all}^{T}W(x_{m},y_{m})RSSI_{all} \end{array}$$

(7) $$X_{all}=\left[\begin{array}{lll} 1 & X_{11} & X_{12}\\ 1 & X_{21} & X_{22}\\ 1 & \text{⁞} & \text{⁞}\\ 1 & X_{j1} & X_{j2} \end{array}\right]\;RSSI_{all}=\left[\begin{array}{c} RSSI_{1}\\ RSSI_{2}\\ \text{⁞}\\ RSSI_{j} \end{array}\right]\;j=1,2,...,R$$

$RSSI_{j}$ is the RSSI value from $RSSI_{AP}(i,j)$ or $RSSI_{A\text{nchor}}(i,j)$.

Therefore, the RSSI $\bar{RSSI}(x_{m},y_{m})$ at location $\left(x_{m},y_{m}\right)$ can be obtained in Equation (9). $X_{m}$ is the parameter matrix at location *m*:

(8) $$\begin{array}{l} X_{m}=[1\;\log(d_{m}/d_{o})\;\exp(\log(d_{m}/d_{0})/\log e)\;]\\ d_{m}=\sqrt{{\left(x_{m}-x_{APi}\right)}^{2}+{\left(y_{m}-y_{APi}\right)}^{2}} \end{array}$$

(9) $$\bar{RSSI}(x_{m},y_{m})=X_{m}\beta (x_{m},y_{m})$$

Since the RGWR belongs to the Nadaraya-Watson estimating method, the boundary effect is huge, which will make the coefficient function heavily distorted in the boundary area and lead to poor results. Therefore we propose two virtual anchors for each AP. $Anchor_{A}$ is one meter from the AP and can be measured in advance as $P_{0}$, while $Anchor_{B}$ is at the edge of the AP coverage and the RSSI at $Anchor_{B}$ is set as −92 dBm, which is the detection sensitivity of the AP in our system.

#### 2.2.2. Deployment of the WIFI Anchors

As the fingerprint map is constructed largely based on the information from WIFI anchors, their deployment features, such as the number and location, will seriously affect the system performance.

If the required positioning accuracy is the same in the whole area, the WIFI anchors can be deployed uniformly. Otherwise, the deployment density in the interesting area should be higher. However, at least three calibration points, including APs and WIFI anchors should be in the coverage of each AP to perform RGWR algorithm well.

In the indoor scenario, the WIFI signal propagating character will change seriously when encountering a corner or a larger barrier. The basic mathematics calculation will result in a large error unless an additional WIFI anchor is deployed to indicate the signal profile in the area.

According to the sampling and interpolation theory, the more accurate samples we have for our system, the more reliable the regression performance is. However, we should find the minimum but appropriate number of WIFI anchors for practical use. One method is to ensure that the WIFI anchor can be received by more APs. Consequently, one anchor can be used for the signal regression of several APs.

#### 2.2.3. Updating Radio Map

The initial radio map can be constructed by the RGWR algorithm proposed in Section 2.2.1 for the deployed system. Afterward, the radio map should be self-updated to ensure the positioning performance along with the environment or time changes. As the renovated APs and low-power WIFI anchors exist, the positioning server can monitor the signal changes effectively. The situations of updating database have been divided into the following cases:

Case 1: The change caused over time. The time-varying feature of WIFI signal requires revising the database at different time even in the same scenario, such as the office hours and rush hours.

Case 2: The change caused over environments, such as the moving of furniture, the adding of walls and so on. As the fingerprint positioning method uses the radio feature collected in offline stage for online positioning, the signal propagation model is assumed to be stable.

Case 3: The change caused by APs, that including the AP locations and working status. If one AP is moved, the signal feature in the corresponding areas will change and make the radio database out of date. On the other hand, the breakdown AP would make the fingerprint matching confused.

**Figure 2:**
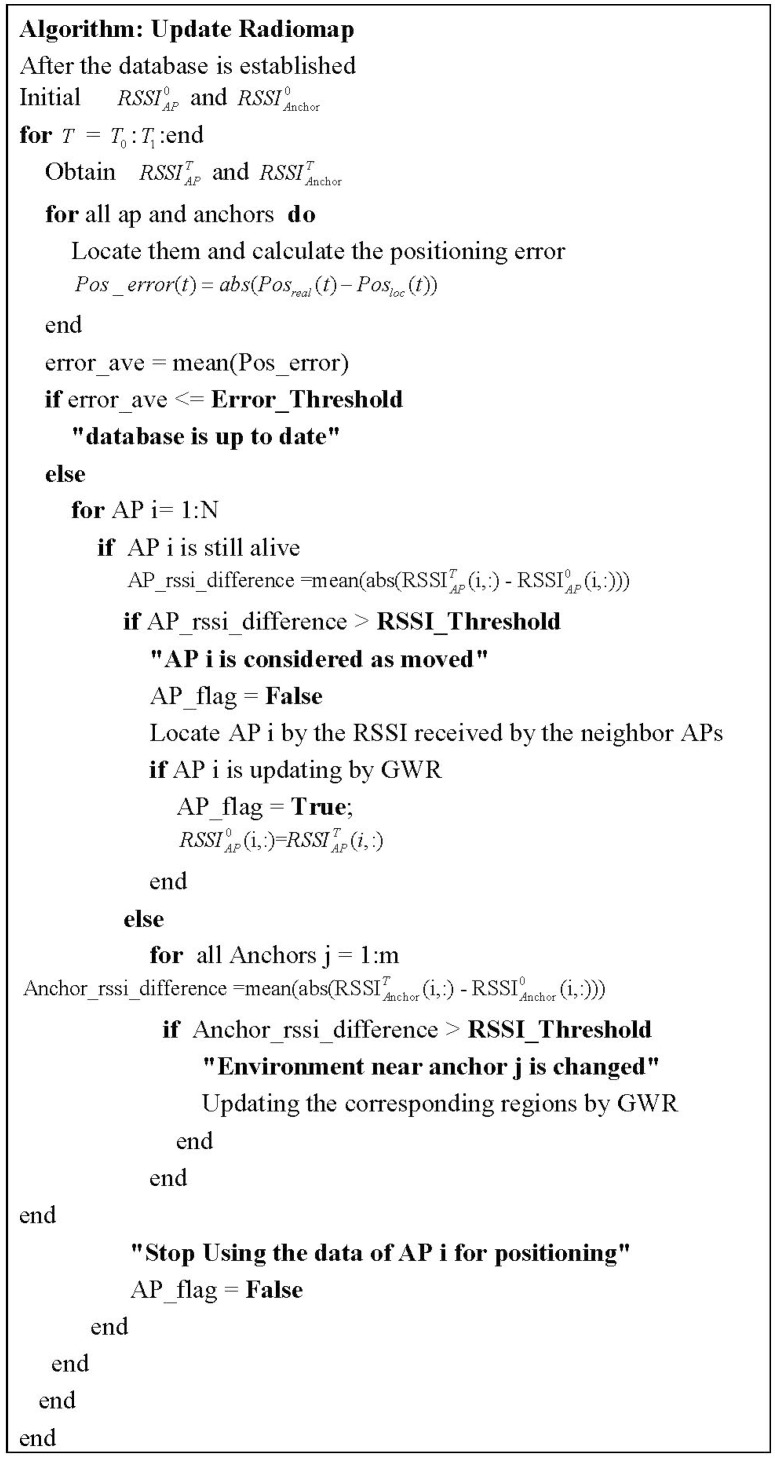
Pseudo-codes algorithm for updating the database.

The three cases of changes above could be dealt with through this proposed system. The positioning server runs the dynamic online-calibrated radio map construction and updating process automatically. The signal features can be obtained in short periods, such as 1 s, and the updating process is triggered. The radio map database updating process is triggered to start, which includes “temporary updating” for time changes, APs’ breakdown, and “permanent updating” for environment changes or APs’ location changes.

The scheme for detecting and triggering the database updating are proposed in pseudo-codes (see Figure 2). $RSSI_{AP}^{T}$ and $RSSI_{anchor}^{T}$ stand for the RSSI vector of each AP and anchor in time T respectively. All the calibration points’ position results $Pos_{loc}$, *i.e.*, the available APs and WIFI anchors will compare to the real locations $Pos_{real}(i)$ to find the average positioning error error_ave to decide finally whether the database is the latest or needs to be updated based on the reference positioning error. If the average RSSI difference in the calibration point AP_rssi_difference is smaller than RSSI_Threshold, the locations of APs are considered not changed and the RGWR algorithm is used for the real time database construction directly. Otherwise, its new position should be recalculated by pattern matching first.

Simultaneously the RSSI of each anchor at time T $RSSI_{A\text{nchor}}^{T}$ is compared to $RSSI_{A\text{nchor}}^{0}$ stored in the database to find whether the environment near that grid changes. The threshold Error_Threshold is set as 6 m and RSSI_Threshold is set as 15 dBm in our systems, both of which can be reconfigured based on the environment or the requirements. RSSI_Threshold stands for a tolerance variation range of RSSI. The value is set based on the WIFI fluctuation variance and the estimated error. Error_Threshold stands for the tolerance threshold of the system. The value is set based on the WIFI fluctuation variance and the inherent positioning error.

### 2.3. Positioning

There are two schemes for online positioning. Most traditional WIFI positioning systems are UE-based [33], which require the UE to locate itself or to send the RSSI report to the positioning server for matching after scanning the surrounding APs. Due to the lack of standardization for hardware and software, different WIFI chipsets, antennas, and encapsulation materials, the RSSI fingerprints collected by different devices may change a lot, which is known as the device diversity problem [33,34]. On the other hand, in the AP-based method the APs can scan all the devices with WIFI modules. Most of the APs in the public areas, such as markets and hospitals, are deployed by the network operator and they have the same model. Moreover, the transmitting power of mobile devices is almost the same, which provides advantages for positioning with AP-based method. However, in the case of applying UE-based method, the transmitting power of APs may change adaptively due to power control, which could be barriers for WIFI positioning. Therefore we adopt the AP-based scheme in our system. The signal attenuation formula is shown in Equation (10), where $P_{r}$ is the signal strength obtained by the AP and $P_{D}$ strands for the factor of WIFI detected module. The transmitting power $P_{T}$ is the same from WIFI devices:

(10) $$P_{r}=P_{T}\text{+}P_{Antenna}-P_{PathLoss}+P_{D}+\phi$$

Thus, the RSSI from each device will only be affected by the random variable ϕ and the antennas of mobile phones $P_{Antenna}$. In order to avoid unexpected positioning process, only after the mobile device sending a positioning request to the server, the positioning calculation would be performed. The widely used K nearest neighbors (KNN) algorithms is used to evaluate the system performance.

## 3. Experiments Section

### 3.1. Experimental Setup

**Figure 3:**
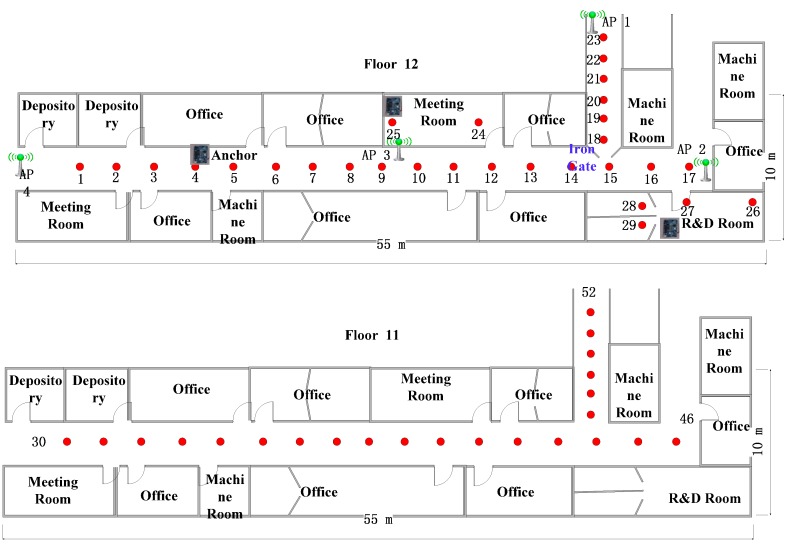
Experiment area (scenario one).

The physical experiments are performed in two scenarios. The first one is the Beijing Tian Chuang Technology office building (Beijing, China). It is approximately 55 m by 10 m (see Figure 3). The environment was deployed by the present authors with four renovated IEEE 802.11WLAN APs (2.4 GHz). In the experiments, MI 2A is used as the test mobile terminal, with Android 4.1.1 system. The system frameworks together with the proposed algorithms are evaluated in the experiments.

### 3.2. RSSI Estimation Accuracy

**Figure 4:**
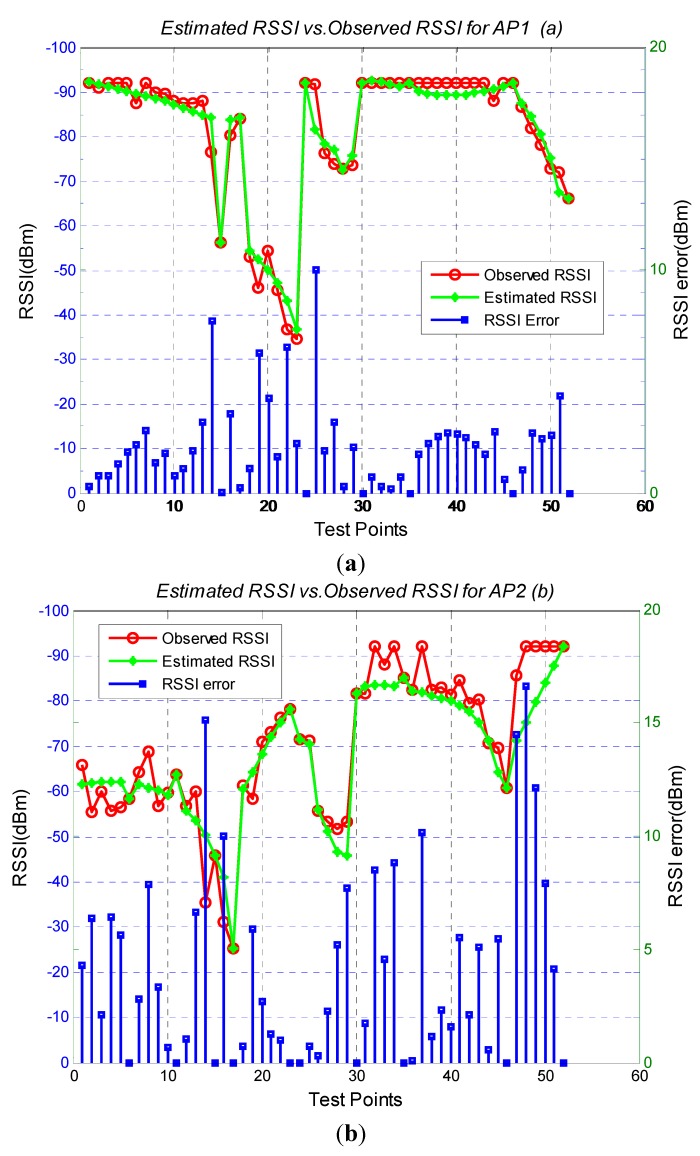

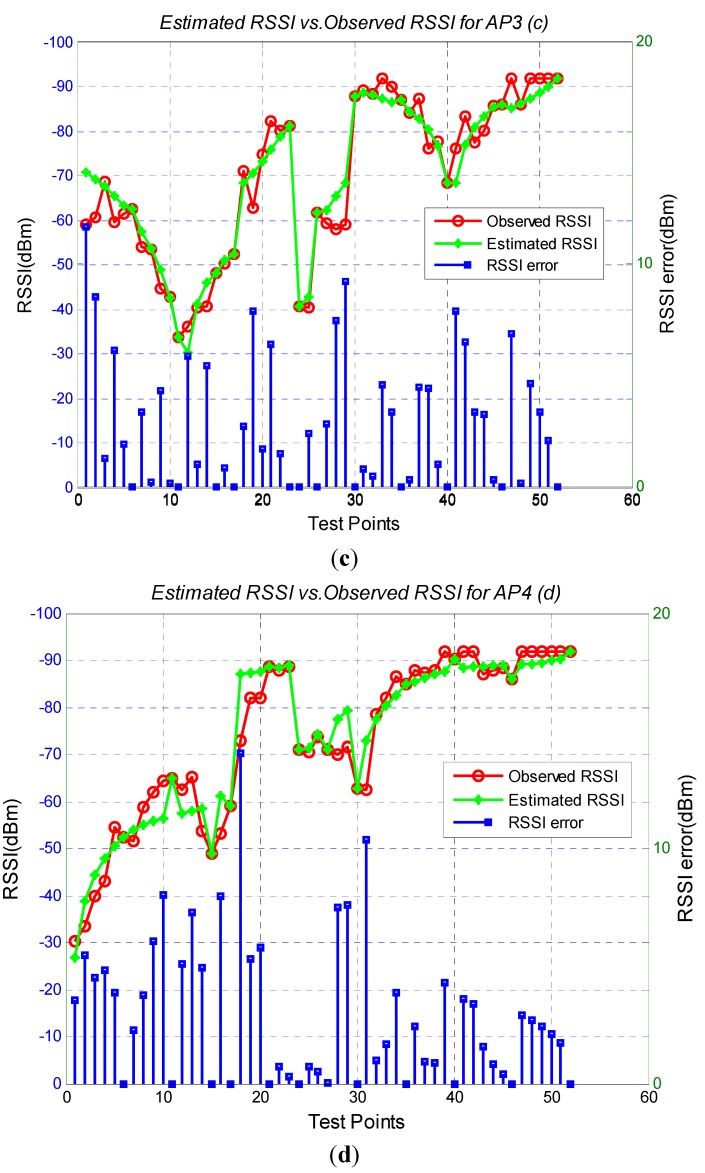
Estimated RSSI errors for testing points in scenario one. (**a**) Estimated Results for AP1; (**b**) Estimated Results for AP2; (**c**) Estimated Results for AP3; (**d**) Estimated Results for AP4

To assess the RSSI estimation accuracy, a total of 52 reference locations are selected in the testing areas (see red dark circles in Figure 3). As the signal attenuates insignificantly with the distance between neighboring grids along the corridor, the area of the virtual grids is set as 3 m × 3 m. We deployed three WIFI anchors, one in the center of the corridor, another one is in the meeting room and last one in the R&D room. Both the information from the APs and WIFI anchors are used in the algorithms. The comparison results are presented in Figure 4, showing that most of the RSSI error between observed RSSI and estimated RSSI is smaller than 10 dBm. The overall results are summarized in Table 3. It is noted that the RSSI estimation accuracy is encouraging, with average error of 3.76 dBm.

**Table 3:** RSSI Errors (dBm) in Testing Points in Scenario one.

	AP1	AP2	AP3	AP4	Average
Scenario one	3.48	4.12	3.66	3.79	3.76

### 3.3. Location Estimation Accuracy

#### Static Test and Dynamic Test

**Figure 5:**
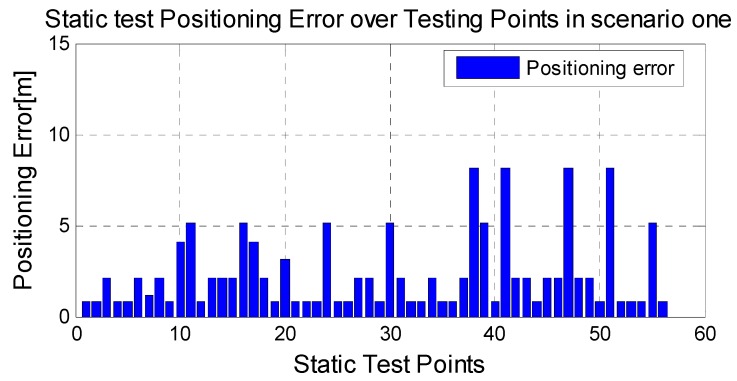
Static Test positioning errors in scenario one with the proposed radio map.

In the static test, the tester stands on the reference point and the observed RSSI values are sent to the positioning server, which uses the recently constructed radio map to provide a location for each reference point. For each reference point in the area, 30 positioning results are obtained and the average error is given in Figure 5, which shows that the average error is 2.4 m with the proposed radio map.

These results are very similar or even slightly better than the results reported in the former papers [13,16,17,18,19]. Our approach has the advantage of not depending on offline training data, which enables it to automatically constructed the positioning system and dynamically model the changes in the environment.

During the dynamic test, a WLAN-enabled mobile device (MI 2A) moves around the experiment area along the known waypoints. The AP scans the signal transmitted by the device and sends the RSSI to the positioning server. At the known waypoints, we stopped and recorded the reference location for positioning error calculation purpose.

**Figure 6:**
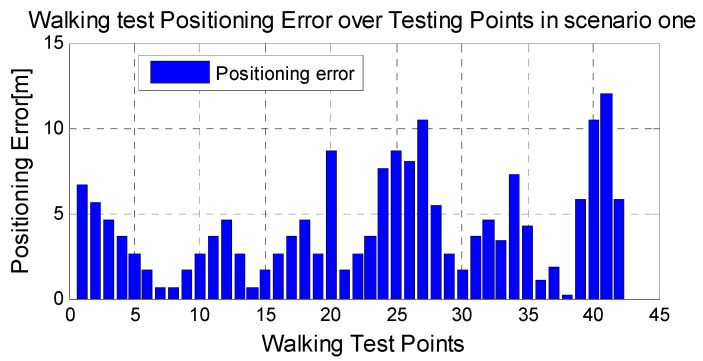
Walking Test positioning errors on Floor 12 with the proposed Radio map.

The average positioning errors of 25 tracks in both scenarios are given in Figure 6, which shows good performance both in the static test and dynamic test with the advantage of removing the time-consuming offline surveying work. Our results can achieve the accuracy level of grid size.

We deploy the WIFI anchors in our experiments above based on the basic principle that at least three calibration points, including APs and WIFI anchors should be in the coverage of each AP to execute the RWGR algorithms. In addition, the corners and interesting rooms are the candidate locations. Take the test points on the 12th floor in scenario one for example, the estimated error of the test points in the meeting room increase to 15 dBm when the WIFI anchor in the room is removed. That illustrates the importance of the WIFI anchors in the rooms.

As shown in Figure 5 and Figure 6, the RSSI error is comparable lower at the points near the WIFI anchors. Consequently, the positioning error is also smaller near the anchors. It is concluded that the WIFI anchors provide positive effect for the neighboring area.

To find out the relationship between the anchor number and the average positioning error, we decrease the number of WIFI anchors one by one in scenario one. The results are shown in Table 4, which indicates that the WIFI anchors are significant for the positioning performance. The more WIFI anchors, the smaller the average positioning error in our experiments. The detailed principles for the WIFI anchors deployment are shown in the following scenario.

**Table 4:** Average positioning error *vs.* Anchor number in scenario one (MI 2A).

Number of Anchors	0	1	2	3
Static Test(m)	8.3 m	5.3 m	4.0 m	2.4 m
Walking Test(m)	9.2 m	6.8 m	5.9 m	4.2 m

### 3.4. Solution for the Changes Caused by Time and Environment

The radio map changes caused by time are always a serious problem in WIFI positioning systems. Though several specific radio maps created at different times could be stored in the positioning server, such as 8 a.m., 11 a.m., and 5 p.m., the cost is too large. In addition, most existing algorithms cannot solve the RSSI changes due to the environment.

**Figure 7:**
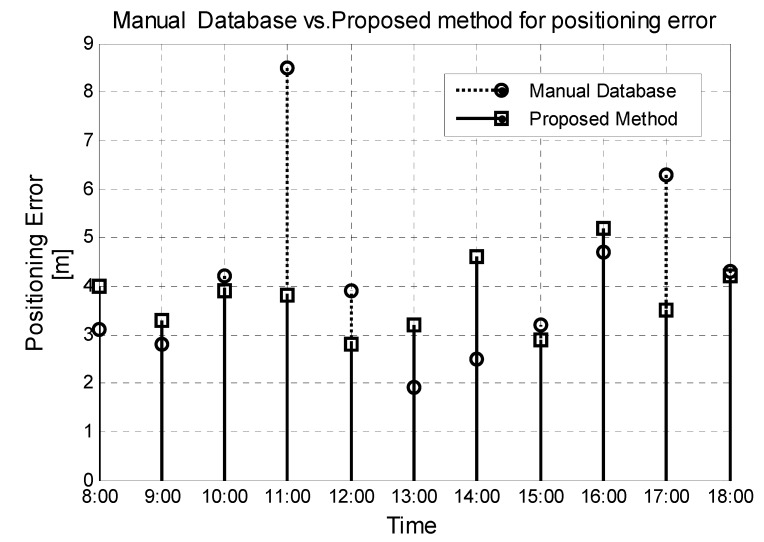
Comparison between the manual database and proposed method at different time.

Four mobile phones are placed uniformly in fixed locations on the 12th floor in scenario one. During every hour between 8:00 and 18:00, we can obtain 20 positioning results for each mobile phone using the manual database and the newly developed database with our proposed method, respectively. The manual database is collected at 9:00 and remains unchanged. The average positioning errors are show in Figure 7. In the common work time, the performance of the traditional database is a little better than our proposed method. However, the traditional method cannot manage the situation, when many people walk in the corridor and the WIFI signal is affected seriously during the rest time at around 11:00. Moreover, when we move the AP3 to another place at 17:00, the positioning error obtained from the manual database increases greatly and the superiority of our method is obvious.

**Figure 8:**
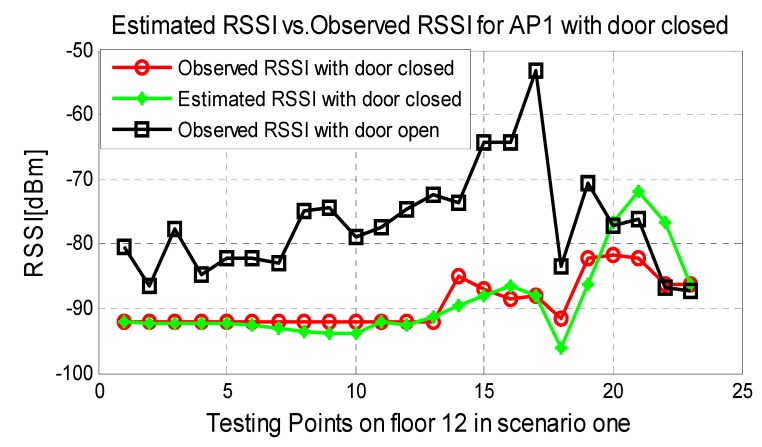
Estimated RSSI for AP1 with door closed.

**Figure 9:**
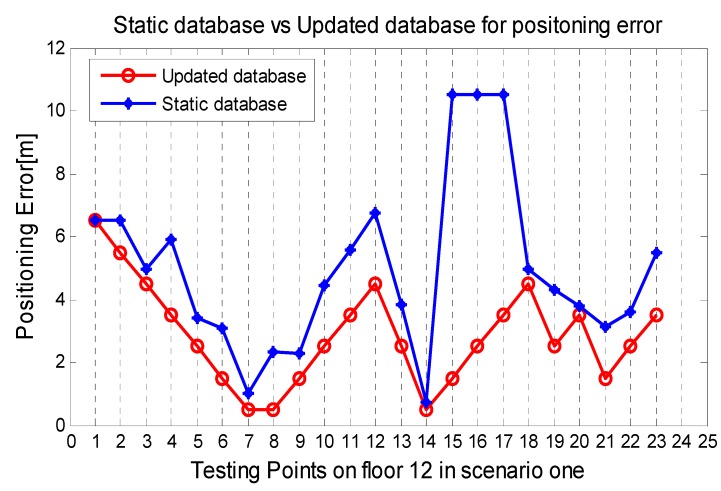
Comparison between the static database and updated database for positioning error with door closed.

To verify the self-updating function of our database, an iron door near the 18th reference points was opened and closed to simulate an environment change (see Figure 3) in scenario one. AP1 will be triggered by our proposed algorithm and switches the AP_flag from True to False before the updating process is completed. Compared the manual database with the updated database in Figure 8, the difference caused by the closed door is 15–25 dB and the updating process are accurate and essential. Consequently, the results in Figure 9 show that the positioning performance can be guaranteed with the database updated by the propose method when the door is closed. Additionally, if one of the APs is broken down, the system will also detect the accident immediately by the RSSI matrix $RSSI_{AP}$ and switch the flag of that AP from True to False. Then, only the APs with True flag are used in the positioning.

### 3.5. Further Evaluation

**Figure 10:**
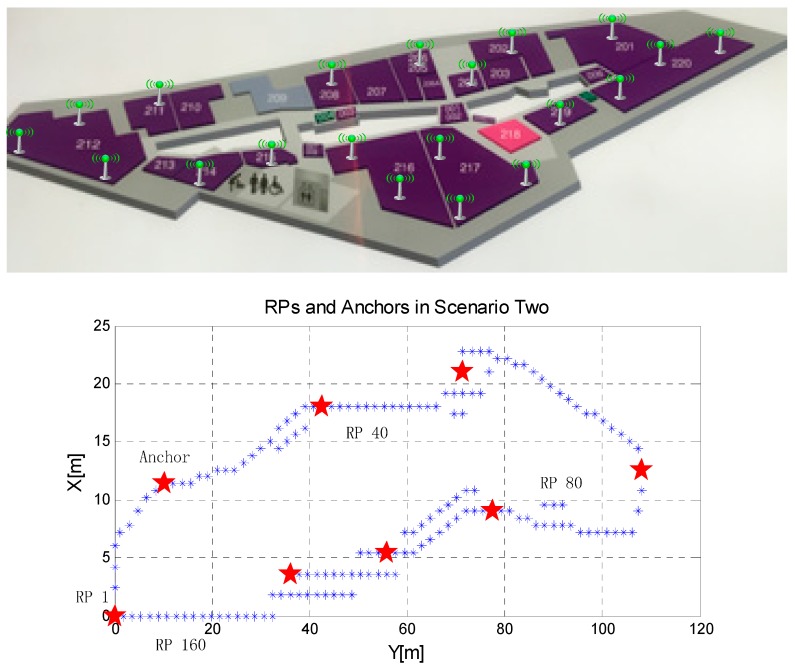
Experiment area (scenario two).

To further evaluate our proposed GWR algorithms, another experiment with a bigger and more complicated environment is performed. The second environment is 25 m by 115 m on the second floor in the Zhong Guan Cun shopping center in Beijing (see Figure 10). There are 20 traditional IEEE 802.11WLAN APs (2.4 GHz) units deployed by the shopping center which already existed in the environment. As our proposed system constructing frameworks has been validated in scenario one, this experiment is performed to further evaluate the proposed GWR algorithm and analyze how the anchors affect the system performance. We divide the area into 163 small areas of 1.5 m × 1.5 m with one reference point in each small area, the same as the size of floor tiles. Eight anchors with the function of receiving RSSIs from APs are uniformly deployed in the locations shown in Figure 10.

**Figure 11:**
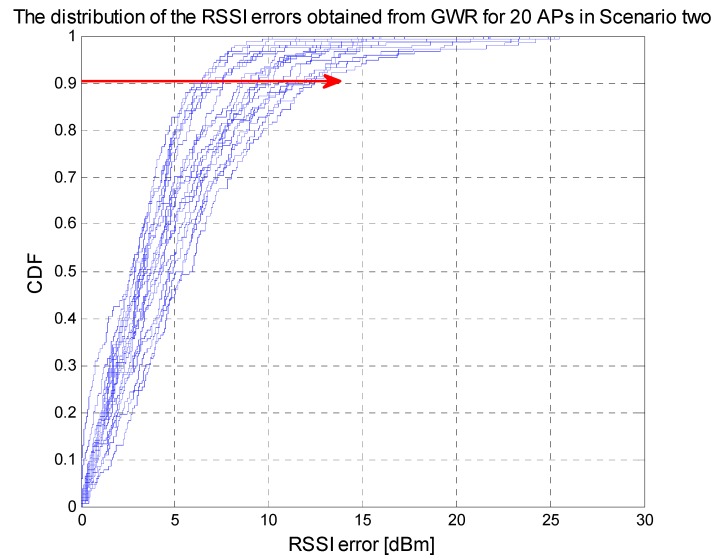
Estimated RSSI errors for testing points in scenario two.

As shown in Figure 11, there are 20 cumulative distribution function curves which stand for the estimated RSSI errors of each AP at all the reference points, and 30 RSSI results are obtained for every reference point. 90% of the estimated errors are smaller than 12 dBm of all the APs and the average estimated error of each AP are between 3.18 dBm and 6.3 dBm.

To evaluate our proposed GWR algorithm, the RBF based aggregation algorithm [27] is also run for comparison of the performance of average estimated RSSI errors together with the RSSI discrimination at different reference points.

**Figure 12:**
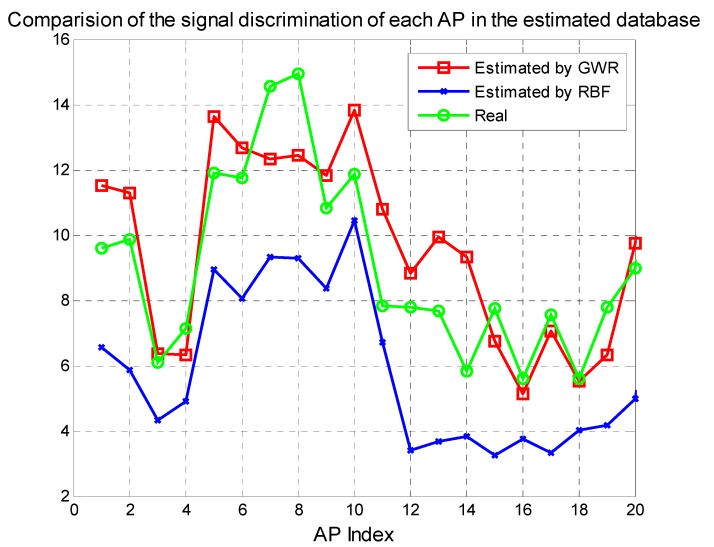
Signal discrimination for RPs in scenario two.

**Figure 13:**
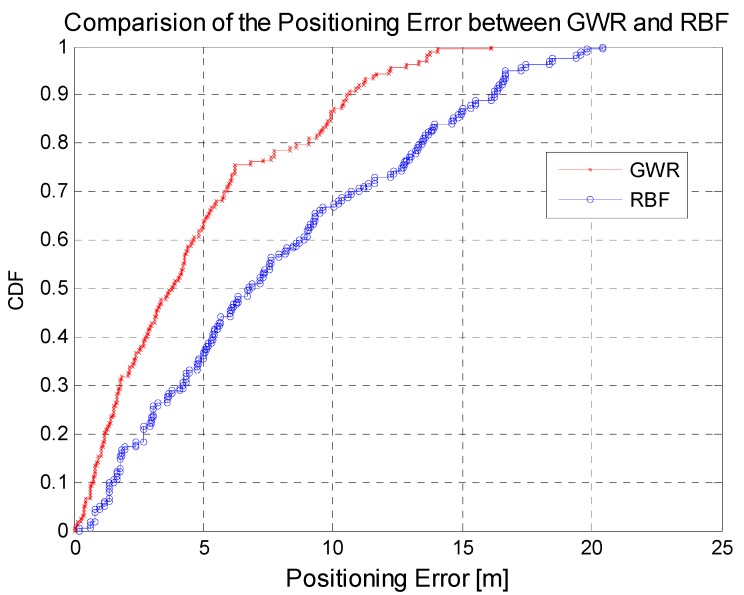
Positioning error based on GWR and RBF in scenario two.

The results in Table 5 show that GWR is much better than RBF in the average RSSI errors. Because there are 163 RPs in scenario two, the improvement of average RSSI errors of 1–2 dBm is encouraging. Furthermore, the signal variance of all the RPs of each AP in Figure 12 shows that the RSSIs estimated by the RBF lost much signal discrimination compared to the true RSSIs, while the performance of the results estimated by GWR just degrades a little. Higher signal discrimination of RPs would result in better positioning results. The cumulative distribution function curves of the walking positioning error in Figure 13 show that the proposed GWR obtains much better positioning results, with an average error of 5.1 m.

**Table 5:** Comparison of average RSSI Errors (dBm) between RBF and GWR in scenario two.

AP Index	1	2	3	4	5	6	7	8	9	10
RBF	4.5	3.9	4.3	4.7	3.8	3.9	5.9	5.7	6.1	4.5
GWR	3.1	3.6	3.6	4.0	3.3	3.4	4.9	5.0	4.2	3.4
AP index	11	12	13	14	15	16	17	18	19	20
RBF	5.9	7.2	5.2	5.1	5.7	7.5	6.3	4.9	5.6	5.2
GWR	4.6	6.0	5.4	5.2	5.2	6.3	5.6	5.2	4.1	3.9

**Figure 14:**
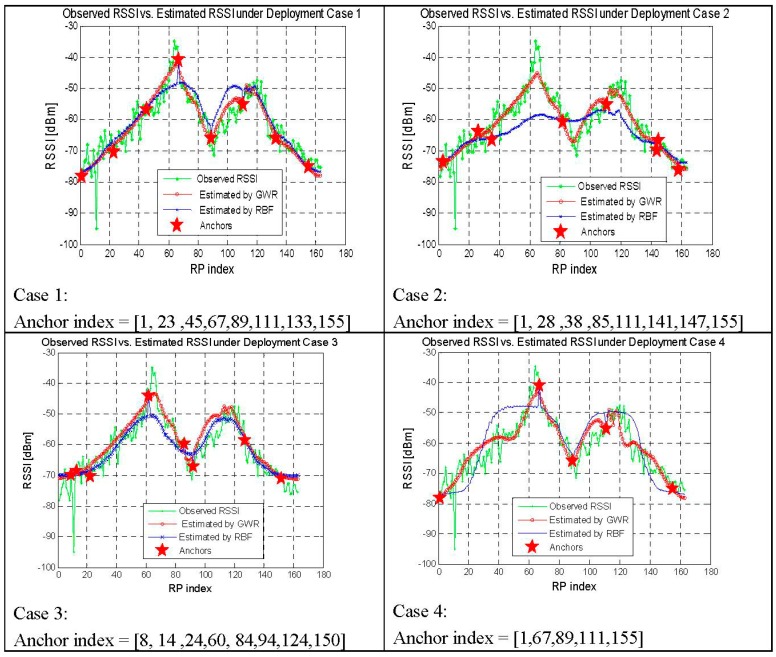
Impact of the anchors in four cases.

To evaluate the impact of anchors, we deploy the anchors in the four cases shown in Figure 14. The RSSI profiles of one AP are demonstrated for the real observed RSSI, the results estimated by GWR and that by RBF. There are eight anchors in case 1, case 2 and case 3, and the results obtained by GWR all show good accuracy. However, the performance of RBF degrades greatly when the anchors in location 64 and 90 of case 1 are moved in case 2, as anchors located just around the characteristic points of the distribution are of the most important. In case 4, only those five anchors in case 1 are retained, both of the two estimating algorithms obtain good RSSI profiles, with just a small increase in the average RSSI error compared with case 1 of eight anchors. A conclusion from the above results can be drawn that the performance of our proposed GWR algorithm is more robust under the conditions of approximately uniform and full coverage deployments. Furthermore, the anchors should be deployed on the locations that stand for the features of RSSI profiles, such as the nearby area of AP (normally the point of larger RSSI), the corner of the blocked area and the far away area from the AP, to obtain better performance.

As our proposed algorithm can also be used to obtain the full WIFI database if the data of manual sampling are treated as the data from the anchors, the conclusion is also important. Then, only a few manual samples are required as virtual anchors.

## 4. Conclusions

In this paper, a novel method RGWR for the calibration of WIFI fingerprint databases is proposed. The basic advantage of the proposed method is that it uses the online RSSI information from the new renovated WLAN AP and anchors to dynamically construct a fine radio map that accurately models the signal power distribution over the whole environment without time-consuming offline surveys. The system can also deal with the changes caused by time and environment together with the detection and updating scheme as proposed. The proposed algorithms provide better performance over the RBF algorithm in the average estimated RSSI error, the RSSI discrimination of reference points and the average positioning error. The effects of anchors on system performance are further researched. A conclusion can be drawn that the anchors should be deployed on the locations that stand for the features of RSSI profiles to obtain better performance.

The positioning error of our newly constructed system is 2–3 m and 4–5 m for static and walking scenarios, respectively, and it is among the most accurate results currently obtained by systems with little human intervention. The accuracy of our system is acceptable for most indoor applications. What’s more, the proposed system is easy to be established and maintain for wide deployment. If higher accuracy is required, we can place more WIFI anchors or collect some fingerprints manually in sparse grids as additional input to the RGWR algorithm. In the future, we will perform further research and experiments on the deployment of the WIFI anchors, and potential applications for scenarios like airports and markets.

## References

[1] Gu Y., Lo A., Niemegeers I. (2009). A survey of indoor positioning systems for wireless personal networks. IEEE Commun. Surv. Tutor..

[2] Hui L., Darabi H., Banerjee P., Jing L. (2007). Survey of Wireless Indoor Positioning Techniques and Systems. IEEE Trans. Syst. Man Cybern. Part C.

[3] Mahtab Hossain A., Jin Y., Soh W.-S., Van H.N. (2013). SSD: A robust RF location fingerprint addressing mobile devices heterogeneity. IEEE Trans. Mob. Comput..

[4] Kjargaard M.B. (2011). Indoor location fingerprinting with heterogeneous clients. Pervasive Mob. Comput..

[5] Yu K., Dutkiewicz E. (2013). NLOS identification and mitigation for mobile tracking. IEEE Trans. Aerosp. Electron. Syst..

[6] Mihaylova L., Angelova D., Bull D., Canagarajah N. (2011). Localization of mobile nodes in wireless networks with correlated in time measurement noise. IEEE Trans. Mob. Comput..

[7] Fang S., Wang C., Tsao Y. (2014). Compensating for Orientation Mismatch in Robust WIFI Localization Using Histogram Equalization. IEEE Trans.Veh. Technol..

[8] Liu J., Chen R., Pei L., Guinness R., Kuusniemi H. (2012). A Hybrid Smartphone Indoor Positioning Solution for Mobile LBS. Sensors.

[9] Au A.W.S., Chen F., Valaee S., Reyes S., Sorour S., Markowitz S.N., Gold D., Gordon K., Eizenman M. (2013). Indoor Tracking and Navigation Using Received Signal Strength and Compressive Sensing on a Mobile Device. IEEE Trans. Mob. Comput..

[10] MikkelBaun K., Valdemar M.C. Hyperbolic Location Fingerprinting: A Calibration-free Solution for Handling Differences in Signal Strength (concise contribution). Proceedings of the IEEE Sixth Annual IEEE International Conference on Pervasive Computing and Communications.

[11] Chai X., Yang Q. (2007). Reducing the calibration effort for probabilistic indoor location estimation. IEEE Trans. Mob. Comput..

[12] Wang H., Ma L., Xu Y., Deng Z. Dynamic Radio Map Construction for WLAN Indoor Location. Proceedings of the 2011 International Conference on Intelligent Human-Machine Systems and Cybernetics (IHMSC).

[13] Lee M., Han D. QRLoc: User-involved Calibration Using Quick Response Codes for Wi-Fi Based Indoor localization. Proceedings of the 2012 7th International Conference on Computing and Convergence Technology (ICCCT).

[14] Bisio I., Cerruti M., Lavagetto F., Marchese M., Pastorino M., Randazzo A., Sciarrone A. (2014). A Training less WIFI Fingerprint Positioning Approach Over Mobile Devices. IEEE Antennas Wirel. Propag. Lett..

[15] Shih C.Y., Chen L.H., Chen G.H., Wu E.K., Jin M.H. Intelligent Radio Map Management for Future WLAN Indoor Location Fingerprinting. Proceedings of the Wireless Communications and Networking Conference (WCNC).

[16] Lesser A.M., Okoniewski M., Nielsen J. A Modified Fingerprinting Technique for an Indoor, Range-free, Localization system with Dynamic Radio Map Annealing over Time. Proceedings of the 2012 IEEE International Conference on Wireless Information Technology and Systems (ICWITS).

[17] Cai X., Chen L., Chen G. Constructing Adaptive Indoor Radio Maps for Dynamic Wireless Environments. Proceedings of the 2013 IEEE 10th International Conference on Ubiquitous Intelligence and Computing, and 10th International Conference on Autonomic and Trusted Computing (UIC/ATC).

[18] Song L., Chen Y., Trappe W., Greenstein L.J. Non-interactive Localization of Cognitive Radios Based on Dynamic Signal Strength Mapping. Proceedings of the IEEE Sixth International Conference on Wireless on-Demand Network Systems and Services.

[19] Liu X., Zhang S., Lu H., Lin X. (2010). Method for Efficiently Constructing and Updating Radio Map of Fingerprint Positioning. Proceedings of the IEEE GLOBECOM Workshops (GC Workshps).

[20] Kim Y., Chon Y., Cha H. (2012). Smartphone-based collaborative and autonomous radio fingerprinting. IEEE Trans. Syst. Man Cybern. Part C.

[21] Bolliger P., Partridge K., Chu M. (2009). Improving location fingerprinting through motion detection and asynchronous interval labeling. Location and Context Awareness.

[22] Sorour S., Lostanlen Y., Valaee S. Reduced-effort Generation of Indoor Radio Maps Using Crowdsourcing and Manifold Alignment. Proceedings of the IEEE 2012 Sixth International Symposium on Telecommunications (IST).

[23] Atia M.M., Noureldin A., Korenberg M.J. (2013). Dynamic Online-Calibrated Radio Maps for Indoor Positioning in Wireless Local Area Networks. IEEE Trans. Mob. Comput..

[24] Ahn H., Yu W. (2009). Environmental-Adaptive RSSI-Based Indoor Localization. IEEE Trans. Autom. Sci. Eng..

[25] Ni L., Liu Y., Lau Y.C., Patil A. Landmarc: Indoor Location Sensing Using Active RFID. Proceedings of the IEEE International Conference on Pervasive Computing and Communications (PerCom’03).

[26] Krishnan P., Krishnakumar A., Ju W.-H., Mallows C., Gamt S. A System for Lease: Location Estimation Assisted by Stationary Emitters for Indoor rf Wireless Networks. Proceedings of the IEEE Conference on Computer Communications (INFOCOM’04).

[27] Yin J., Yang Q., Ni L. (2008). Learning adaptive temporal radio maps for signal-strength-based location estimation. IEEE Trans. Mob. Comput..

[28] Sun Z., Chen Y., Qi J., Liu J. Adaptive Localization through Transfer Learning in Indoor wi-fi Environment. Proceedings of the Seventh International Conference on Machine Learning and Applications (ICMLA’08).

[29] Wang Q., Chen X., Chen R., Chen Y., Zhang X. (2013). Electromyography-Based Locomotion Patterns Classification and Personal Positioning Toward Improved Context-Awareness Applications. IEEE Trans. Syst. Man Cybern.-Syst..

[30] Fotheringham A.S., Brunsdon C., Charlton M. (2002). Geographically Weighted Regression.

[31] Kaya A.O., Greenstein L.J. A New Path Loss Modeling Approach for In-building Wireless Networks. Proceedings of the 2012 IEEE Global Communications Conference (GLOBECOM).

[32] Akiyama T., Teranishi Y., Okamura S., Shimojo S. An Approach for Filtering Inaccurate Access Point Observation Report in WIFI Positioning System. Proceedings of the 2010 IEEE International Conference on P2P, Parallel, Grid, Cloud and Internet Computing (3PGCIC).

[33] Park J.G., Curtis D., Teller S., Ledlie J. Implications of device diversity for organic localization. Proceedings of the 2011 IEEE Proceedings INFOCOM.

[34] Figuera C., Rojo-álvarez J.L., Mora-Jiménez I., Guerrero-Curieses A., Wilby M., Ramos-López J. (2011). Time-space sampling and mobile device calibration for WIFI indoor location systems. IEEE Trans. Mob. Comput..

